# Supplementary material for: Multi‐dataset identification of innovative feature genes and molecular mechanisms in keratoconus
Source: J Cell Mol Med. 2024 Sep 19;28(18):e70079. doi: 10.1111/jcmm.70079 (PMC11412914; doi:10.1111/jcmm.70079)
Supplement: Supplementary file 2 — Material S2. [file JCMM-28-e70079-s002.zip › 7.GSEA analysis/6.SNORA74B/KEGG_TIGHT_JUNCTION.html]

Details for gene set KEGG\_TIGHT\_JUNCTION[GSEA]

|  || Dataset | SNORA74B.group.cls#h\_versus\_l |
| Phenotype | group.cls#h\_versus\_l |
| Upregulated in class | l |
| GeneSet | KEGG\_TIGHT\_JUNCTION |
| Enrichment Score (ES) | -0.40126124 |
| Normalized Enrichment Score (NES) | -1.4668252 |
| Nominal p-value | 0.032323234 |
| FDR q-value | 0.2973236 |
| FWER p-Value | 0.827 |
Table: GSEA Results Summary

  

Fig 1: Enrichment plot: KEGG\_TIGHT\_JUNCTION      
 Profile of the Running ES Score & Positions of GeneSet Members on the Rank Ordered List

  

| SYMBOL | TITLE | RANK IN GENE LIST | RANK METRIC SCORE | RUNNING ES | CORE ENRICHMENT || 1 | CLDN22 | na | 192 | 0.783 | 0.0315 | No |
| 2 | CLDN5 | na | 1859 | 0.384 | 0.0015 | No |
| 3 | MYH11 | na | 2326 | 0.345 | 0.0044 | No |
| 4 | PRKCG | na | 2539 | 0.330 | 0.0139 | No |
| 5 | EPB41 | na | 4166 | 0.254 | -0.0211 | No |
| 6 | RAB3B | na | 4338 | 0.248 | -0.0143 | No |
| 7 | MYH15 | na | 4464 | 0.244 | -0.0063 | No |
| 8 | PRKCQ | na | 5170 | 0.220 | -0.0163 | No |
| 9 | OCLN | na | 6320 | 0.182 | -0.0409 | No |
| 10 | MYL5 | na | 6916 | 0.165 | -0.0503 | No |
| 11 | SYMPK | na | 7080 | 0.160 | -0.0474 | No |
| 12 | GNAI3 | na | 7691 | 0.144 | -0.0583 | No |
| 13 | CLDN23 | na | 7699 | 0.143 | -0.0517 | No |
| 14 | AMOTL1 | na | 7829 | 0.140 | -0.0488 | No |
| 15 | ASH1L | na | 7848 | 0.140 | -0.0427 | No |
| 16 | EXOC3 | na | 7874 | 0.139 | -0.0368 | No |
| 17 | CLDN20 | na | 8410 | 0.127 | -0.0463 | No |
| 18 | CLDN7 | na | 8863 | 0.117 | -0.0538 | No |
| 19 | EPB41L1 | na | 8905 | 0.116 | -0.0495 | No |
| 20 | AKT2 | na | 9380 | 0.105 | -0.0583 | No |
| 21 | KRAS | na | 9766 | 0.097 | -0.0648 | No |
| 22 | CSNK2A1 | na | 9893 | 0.095 | -0.0639 | No |
| 23 | MYH14 | na | 9976 | 0.093 | -0.0619 | No |
| 24 | EXOC4 | na | 10236 | 0.088 | -0.0652 | No |
| 25 | SPTAN1 | na | 10626 | 0.081 | -0.0726 | No |
| 26 | TJP2 | na | 10909 | 0.076 | -0.0772 | No |
| 27 | TJP1 | na | 11082 | 0.073 | -0.0787 | No |
| 28 | PPP2R2D | na | 11122 | 0.073 | -0.0763 | No |
| 29 | LLGL2 | na | 11182 | 0.072 | -0.0746 | No |
| 30 | MYH13 | na | 11208 | 0.071 | -0.0720 | No |
| 31 | MPDZ | na | 11366 | 0.069 | -0.0733 | No |
| 32 | ACTB | na | 11421 | 0.068 | -0.0716 | No |
| 33 | PPP2R2C | na | 11439 | 0.068 | -0.0689 | No |
| 34 | CLDN3 | na | 11534 | 0.066 | -0.0684 | No |
| 35 | EPB41L3 | na | 11577 | 0.066 | -0.0665 | No |
| 36 | PPP2R1B | na | 12000 | 0.060 | -0.0759 | No |
| 37 | PRKCZ | na | 12067 | 0.059 | -0.0750 | No |
| 38 | ACTN4 | na | 12377 | 0.055 | -0.0813 | No |
| 39 | CLDN19 | na | 12660 | 0.051 | -0.0871 | No |
| 40 | CLDN16 | na | 12743 | 0.050 | -0.0871 | No |
| 41 | PTEN | na | 12756 | 0.050 | -0.0851 | No |
| 42 | YBX3 | na | 13049 | 0.046 | -0.0913 | No |
| 43 | CLDN8 | na | 13125 | 0.045 | -0.0913 | No |
| 44 | CTTN | na | 13315 | 0.044 | -0.0947 | No |
| 45 | PARD6G | na | 13697 | 0.039 | -0.1039 | No |
| 46 | CGN | na | 14392 | 0.031 | -0.1224 | No |
| 47 | GNAI2 | na | 14445 | 0.031 | -0.1225 | No |
| 48 | CLDN9 | na | 14622 | 0.029 | -0.1262 | No |
| 49 | MYH2 | na | 14955 | 0.025 | -0.1346 | No |
| 50 | CRB3 | na | 14958 | 0.025 | -0.1335 | No |
| 51 | LLGL1 | na | 15009 | 0.024 | -0.1338 | No |
| 52 | PARD3 | na | 15192 | 0.023 | -0.1380 | No |
| 53 | MYH9 | na | 15415 | 0.020 | -0.1434 | No |
| 54 | PPP2R2B | na | 15631 | 0.018 | -0.1488 | No |
| 55 | MYL12B | na | 16040 | 0.014 | -0.1599 | No |
| 56 | CTNNA1 | na | 16086 | 0.014 | -0.1606 | No |
| 57 | CLDN4 | na | 16601 | 0.008 | -0.1750 | No |
| 58 | PRKCI | na | 16852 | 0.006 | -0.1820 | No |
| 59 | PRKCD | na | 17017 | 0.005 | -0.1865 | No |
| 60 | F11R | na | 17230 | 0.002 | -0.1925 | No |
| 61 | CTNNB1 | na | 17783 | -0.003 | -0.2083 | No |
| 62 | ACTG1 | na | 18268 | -0.009 | -0.2218 | No |
| 63 | RHOA | na | 18290 | -0.009 | -0.2220 | No |
| 64 | AKT1 | na | 18366 | -0.010 | -0.2237 | No |
| 65 | MYL12A | na | 18525 | -0.012 | -0.2277 | No |
| 66 | MAGI3 | na | 18643 | -0.013 | -0.2304 | No |
| 67 | CDC42 | na | 18770 | -0.015 | -0.2334 | No |
| 68 | CTNNA2 | na | 18831 | -0.015 | -0.2344 | No |
| 69 | PRKCH | na | 19480 | -0.023 | -0.2521 | No |
| 70 | MYH3 | na | 19564 | -0.024 | -0.2533 | No |
| 71 | HRAS | na | 19699 | -0.025 | -0.2560 | No |
| 72 | CSNK2B | na | 20420 | -0.033 | -0.2753 | No |
| 73 | RAB13 | na | 20748 | -0.037 | -0.2830 | No |
| 74 | CSNK2A2 | na | 20870 | -0.039 | -0.2846 | No |
| 75 | PPP2R1A | na | 21264 | -0.043 | -0.2939 | No |
| 76 | MYH7 | na | 22198 | -0.054 | -0.3184 | No |
| 77 | PRKCA | na | 22239 | -0.055 | -0.3169 | No |
| 78 | VAPA | na | 22776 | -0.062 | -0.3295 | No |
| 79 | CLDN15 | na | 22839 | -0.063 | -0.3283 | No |
| 80 | CLDN1 | na | 23337 | -0.070 | -0.3393 | No |
| 81 | YES1 | na | 23375 | -0.071 | -0.3371 | No |
| 82 | MYH10 | na | 23780 | -0.077 | -0.3451 | No |
| 83 | CASK | na | 23879 | -0.079 | -0.3442 | No |
| 84 | MYL2 | na | 23893 | -0.079 | -0.3408 | No |
| 85 | CDK4 | na | 24176 | -0.084 | -0.3450 | No |
| 86 | TJP3 | na | 24378 | -0.087 | -0.3467 | No |
| 87 | PPP2R2A | na | 24518 | -0.090 | -0.3465 | No |
| 88 | RRAS | na | 24557 | -0.090 | -0.3433 | No |
| 89 | PPP2CA | na | 25151 | -0.101 | -0.3556 | No |
| 90 | CTNNA3 | na | 25470 | -0.107 | -0.3598 | No |
| 91 | RRAS2 | na | 25730 | -0.112 | -0.3620 | No |
| 92 | PPP2CB | na | 26235 | -0.122 | -0.3708 | No |
| 93 | CLDN6 | na | 26559 | -0.129 | -0.3740 | No |
| 94 | PARD6A | na | 26746 | -0.134 | -0.3731 | No |
| 95 | MYH6 | na | 27723 | -0.158 | -0.3938 | Yes |
| 96 | CLDN18 | na | 27780 | -0.159 | -0.3879 | Yes |
| 97 | MYH1 | na | 27929 | -0.163 | -0.3845 | Yes |
| 98 | NRAS | na | 27969 | -0.164 | -0.3778 | Yes |
| 99 | ACTN1 | na | 28221 | -0.171 | -0.3770 | Yes |
| 100 | TJAP1 | na | 28278 | -0.172 | -0.3705 | Yes |
| 101 | GNAI1 | na | 28399 | -0.176 | -0.3656 | Yes |
| 102 | CLDN11 | na | 28639 | -0.183 | -0.3638 | Yes |
| 103 | MYH4 | na | 28706 | -0.186 | -0.3569 | Yes |
| 104 | MYH7B | na | 29317 | -0.204 | -0.3649 | Yes |
| 105 | SRC | na | 30227 | -0.234 | -0.3801 | Yes |
| 106 | ACTN2 | na | 30345 | -0.239 | -0.3722 | Yes |
| 107 | MYL9 | na | 30587 | -0.249 | -0.3674 | Yes |
| 108 | CLDN17 | na | 31516 | -0.295 | -0.3802 | Yes |
| 109 | MAGI1 | na | 31942 | -0.320 | -0.3774 | Yes |
| 110 | CLDN10 | na | 31975 | -0.322 | -0.3631 | Yes |
| 111 | MYH8 | na | 32609 | -0.361 | -0.3643 | Yes |
| 112 | PARD6B | na | 32690 | -0.367 | -0.3492 | Yes |
| 113 | CLDN14 | na | 32746 | -0.372 | -0.3332 | Yes |
| 114 | ACTN3 | na | 33200 | -0.413 | -0.3267 | Yes |
| 115 | EPB41L2 | na | 33227 | -0.416 | -0.3078 | Yes |
| 116 | MYL7 | na | 33280 | -0.422 | -0.2893 | Yes |
| 117 | IGSF5 | na | 33399 | -0.438 | -0.2720 | Yes |
| 118 | HCLS1 | na | 33522 | -0.452 | -0.2541 | Yes |
| 119 | PRKCB | na | 33882 | -0.508 | -0.2405 | Yes |
| 120 | MYL10 | na | 34073 | -0.549 | -0.2200 | Yes |
| 121 | MAGI2 | na | 34171 | -0.573 | -0.1957 | Yes |
| 122 | JAM2 | na | 34268 | -0.600 | -0.1701 | Yes |
| 123 | PRKCE | na | 34306 | -0.615 | -0.1420 | Yes |
| 124 | CLDN2 | na | 34525 | -0.714 | -0.1145 | Yes |
| 125 | MRAS | na | 34571 | -0.753 | -0.0802 | Yes |
| 126 | AKT3 | na | 34657 | -0.880 | -0.0410 | Yes |
| 127 | JAM3 | na | 34668 | -0.909 | 0.0017 | Yes |
Table: GSEA details [plain text format]

  

Fig 2: KEGG\_TIGHT\_JUNCTION      
 Blue-Pink O' Gram in the Space of the Analyzed GeneSet

  

Fig 3: KEGG\_TIGHT\_JUNCTION: Random ES distribution      
 Gene set null distribution of ES for **KEGG\_TIGHT\_JUNCTION**
